# Fuzzy Traffic Control with Vehicle-to-Everything Communication

**DOI:** 10.3390/s18020368

**Published:** 2018-01-27

**Authors:** Muntaser A. Salman, Suat Ozdemir, Fatih V. Celebi

**Affiliations:** 1 Department of Information Systems, College of Computer Sciences and Information Technology, University of Anbar, 55431 Baghdad, 55 Ramadi, Anbar, Iraq; 2 Computer Engineering Department, Ankara Yildirim Beyazit University, 06010 Ankara, Turkey; fvcelebi@ybu.edu.tr; 3 Computer Engineering Department, Gazi University, 06570 Ankara, Turkey; suatozdemir@gazi.edu.tr

**Keywords:** traffic signal control, V2X communication, intersection, fuzzy system, acceleration and stopped delay, traffic policy

## Abstract

Traffic signal control (TSC) with vehicle-to everything (V2X) communication can be a very efficient solution to traffic congestion problem. Ratio of vehicles equipped with V2X communication capability in the traffic to the total number of vehicles (called penetration rate PR) is still low, thus V2X based TSC systems need to be supported by some other mechanisms. PR is the major factor that affects the quality of TSC process along with the evaluation interval. Quality of the TSC in each direction is a function of overall TSC quality of an intersection. Hence, quality evaluation of each direction should follow the evaluation of the overall intersection. Computational intelligence, more specifically swarm algorithm, has been recently used in this field in a European Framework Program FP7 supported project called COLOMBO. In this paper, using COLOMBO framework, further investigations have been done and two new methodologies using simple and fuzzy logic have been proposed. To evaluate the performance of our proposed methods, a comparison with COLOMBOs approach has been realized. The results reveal that TSC problem can be solved as a logical problem rather than an optimization problem. Performance of the proposed approaches is good enough to be suggested for future work under realistic scenarios even under low PR.

## 1. Introduction

Traffic signal control can be self-organizing and adaptable to traffic condition changes by using vehicular ad hoc network (VANET) technology and an intelligent control algorithm. VANET technology provides extensive information of approaching vehicles for an intersection. Vehicles frequently transmit specific messages (e.g., basic safety message (BSM) in United States (US), or cooperative awareness message (CAM) in European countries (EU)), containing all required relevant information (e.g., speed, position ... etc.). A traditional method to acquire data from VANETs is to receive messages with a central infrastructure called roadside unit (RSU). After receiving the message, RSU extract the most important information that is suitable for TSC. This extraction depends on the message’s information and what does it represent (i.e., traffic monitoring). Then, controlling an intersection through TSC required an algorithm that can deal with the RSU’s information sufficiently.

In US and EU, several researches have been conducted for TSC of an intersection using vehicular communication protocols. Generally speaking, based on PR, two different approach for TSC can be observed. Either Full Penetration Rate (FPR) or Partial Penetration Rate (PPR).

FPR-based approach is built upon an assumption that all vehicles communicate with each other for compressing the description about the traffic conditions and give a suitable decision for TSC. With this approach, traffic condition indicators are able to determine without involving an RSU. Hence, it may be applied for virtual TSC (or Virtual Traffic Light VTL) of an intersection without real traffic signals infrastructure. In this context, VTL project [1] explored the benefits of vehicle-to-vehicle (V2V) communication for VTL using elected leader. The responsibility of creating the VTL and broadcasting the traffic signal messages is assigned. The main drawback for this approach is the assumption of full PR. Although VTL project contributors in [2] show that VTLs can offer benefits in both throughput and delay with partial deployment scenarios, they are based on the assumption that an external representation is required for the VTL. In this scheme, visibility and legislation aspects were not addressed. Hence, a deployment issue is grown in deciding what is the most appropriate representation for such assumption. Another attempt done in [3] to overcome the issue of PPR using game theory approach. Its results show that by using a dynamic strategy with the penetration ratio, one can provide strong incentives to drivers to adopt VTL technology. A decentralized algorithm for VTL applications, based on IEEE 802.11p V2V communications had also been proposed in [4]. The algorithm has been designed for those intersections where the deployment of traffic lights is not cost-effective. The algorithm effectiveness was validated based on open source software and low-cost hardware with wireless communication interface. In other study [5], the applicability of an in-vehicle traffic light system to assist drivers in passing through un-signalized intersections was tested. Specifically, a driving simulator to analyses the influences of the proposed system on driving operations and eye-gaze behaviors was used.

On the contrary, PPR-based approach is built upon an assumption that some vehicles are capable of communicate with each other and the available traffic signals infrastructure involve RSU. With PPR approach, to overcome the partial knowledge of traffic conditions, TSC can become more beneficial with an intelligent algorithm. In that time, the idea of self-organizing algorithm is merged in 2005 by Gershenson [6]. It proves that using simple rules, traffic signals are able to be self-organizing and adaptable to traffic condition changes. This is considered without direct communication among intersections. In the same paper, a simple self-organizing traffic signals algorithm proposed. The mean idea of which is to give preference to vehicles that have been waiting longer and to larger groups of vehicles (called platoons). These groups of vehicles (i.e., platoons) affect the behavior of traffic signals, made them to turn green based on demand.

Recently, the idea of exploiting VANET technology merged into the field of self-organizing TSC. For instance, in [7], a decentralized adaptive TSC algorithm using vehicle to infrastructure (V2I) communication was developed. Their algorithm was phase based and the objective was to minimize total queue length. Hence, TSC problem considered as an optimization problem and was solved by dynamic programming. In [8], a platoon based TSC algorithm under VANET environment was proposed. In this algorithm, two stages were served; standing queue of vehicles and vehicles approaching the intersection. The idea is that the travel time of vehicles can be obtained based on their location and speed. Results showed that the proposed algorithm was efficient even at 25% PR. In [9] a TSC framework for multi-modal under V2I communication was proposed. It was a platoon based algorithm solved for an optimal signal plan based on controller status, priority requests, platoon data and current traffic condition. In [10], another TSC algorithm under VANET environment was also presented. In this algorithm, a kalman filter to estimate cumulative travel time was applied under low PR. Next phase was set to the highest combined travel time and at least 30% PR was required to achieve good results. In [11], a predictive microscopic simulation algorithm for TSC was proposed. The algorithm predicts the future traffic conditions based on data collected from vehicles within suitable strategy. A rolling horizon strategy was chosen to optimize either delay only or a combination of delay, stops and deceleration. Several PRs were considered in this algorithm based on estimating the states of unequipped vehicles from equipped vehicle states.

Previous researches [6,7,8,9,10,11] showed that the PR was a critical parameter in determining the efficiency of the TSC algorithms. From the literature review, it is clear that there are inefficiencies and trade-offs under different PR (e.g., efficient TSC under VANET environment with traffic conditions estimation) that need to be focus on. In this context, the EU FP7 COLOMBO (2012–2015) project [12] exploits V2X communications in the context of TSC. V2X communications were used to determine traffic surveillance information about local queue length (in proximity of an intersection). Such traffic indicators can be used dynamically to adapt TSC algorithms and timings. The contributors of this project focused on developing TSC algorithms using swarm algorithm under low PR. Since swarm algorithm, like any optimization approach, is time consuming, a TSC based on such algorithm requires more investigation to fill the gap between TSC algorithms and optimization algorithms. 

In this paper, in order to fill this gap, a simple fuzzy logic had been investigated in COLOMBO framework instead of swarm algorithm. This is the main contribution for our paper and its done based on dynamic delay model (i.e., more detailed information) that V2X communication can offer under different PR with fuzzy logic estimation system.

The remainder of this paper is organized as follows. Section 2 presents COLOMBO project efforts in the field of intelligent TSC using V2X communication with low PR. Section 3 describes in details the perspective of the approach proposed in this paper. Section 4 analyzes the proposed approach’s performance and reports the primary simulation results collected so far. Comparison with related works is presented in Section 5. Discussions with on ongoing research and conclusive remarks end the paper.

## 2. COLOMBO’S TSC

This project is dedicated towards making self-organizing traffic lights an effective means for practical TSC [9]. In order to do that, many policies were considered under suitable selection algorithm. The common underlying principle of these policies is that each TSC gets information only on the traffic flow on its incoming and outgoing edges and operates independently of all other TSCs. Ideally, a bird’s eye view of the traffic flow on the incoming and outgoing edges with full (i.e., position, speed and route) information about each vehicle should be available. With this information, TSC knows exactly the traffic condition of each incoming and outgoing edge. Both traditional and emerged VANET systems cannot deliver and process this big data (even with FPR-based approach). This means that TSC algorithms rely on estimates of traffic conditions to divide the arrival flow between signal groups. With PPR-based approach an assumption can be stated here. Absolute numbers (like number of vehicle and sum of stops) can be hardly determined, while averaging measures (like average speed) can be retrieved with a sufficient quality.

In this context, COLOMBO project [12], developed an open source framework and confirm the previous assumption using simulations. COLOMBO framework use swarm intelligence algorithm to estimate an abstract of traffic conditions (called it pheromone) based on average speed and it is derivative. With these pheromones, different TSC methods (i.e., policies such as phase, platoon, marching and congestion policy) were developed. Each policy performs under specific traffic condition and not for others. The following subsection give a brief summary for such policies.

### 2.1. Summary of COLOMBO’s Policies

A set of traffic light control policies were defined and presented in COLOMBO project. For example, phase policy used to end current phase as soon as another one has reached traffic threshold. This ending done under satisfaction of minimum duration constraint of the current stage, where this early ending would not make the TSC switch a lot. This policy designed to handle low-medium traffic conditions. This policy does not end the current stage if there are no cars opposing allowed traffic flow currently performing.

Another policy lets all the vehicles in the current green edges pass the intersection before ending green light. This policy is called platoon policy and designed to handle medium-high traffic conditions. Platoon policy will not switch phase unless green light is requested from another one. Even if there are approaching vehicles, maximum phase duration is taken into account in order to preempt the current phase execution. In case of intense traffic conditions, each phase will be executed for maximum allowed time. The performance of the system greatly impacted by the definition of the maximum allowed time for a phase. Another policy adequate when traffic looks too intense from all incoming edges to take any online decision. This policy is called marching policy and designed to handle very low/high traffic conditions. The approach implemented in COLOMBO project to solve this policy with simple rules is to fall back to a static duration for each phase. Finally, policy used when there may be vehicles waiting in front of the intersection and the outgoing edges are congested. In this case, all input edges are inhibited to avoid what is called gridlocks. The system ends the current phase, executing each stage for their minimum duration time. No other phase is activated when the red-light stage is reached until the congestion has been solved. This policy is called congestion policy and designed to handle congested traffic conditions. To this point, the goal of the policy selection procedure is to select which policy should be executed in the TSC under current traffic conditions.

### 2.2. Selection Procedure of COLOMBO’s Policies

In order to select a suitable policy, a large number of parameters need to be appropriately set by COLOMBO framework to achieve best possible performance. Thus, a parameter tuning optimization approach is performed in this project. This process is time consuming and requires a lot of traffic data to be aggregated in static or dynamic approach to overcome the whole traffic conditions. One drawback of this approach comes from the local traffic conditions sample aggregation areas. The challenge in static approaches is to determine the zone length that is neither too large nor too small. On the contrary, the challenge in dynamic approaches, although these approaches adjust to true traffic conditions, is to build and maintain dynamic groups and group leaders.

On the other hand, in COLOMBO framework, a set of vehicles that are approaching an intersection following a common direction are grouped as an intermediate level of abstraction between the RSU and the multitude of surrounding vehicles. From this grouping; the group leader is chosen so that it can reach all other peers by simple single-hop communication (i.e., central position) and coordinate all group members. This grouping and group leader selection procedures under dynamic VANET environment made the grouping procedure results, with different PRs and traffic conditions, fuzzy and uncertain. Instead of using swarm algorithm to abstract traffic conditions, fuzzy logic had been used in this paper to estimate accumulative delay time with respect to the total travel time.

Such indicators can be directly used for estimating the traffic conditions. This estimation, based on average speed and its derivative, is investigated with different PR for intelligent TSC design.

## 3. PROPOSED TSC

In this paper, we propose to follow PPR-based approach for TSC design with different strategy. This is done by estimating vehicles delay time with respect to vehicles sensed time as a direct indicator for logical TSC design. This strategy uses V2X protocol under COLOMBO framework for average speed determination per moment, to estimate the delay time for each edge per moment. This delay time estimation done locally by the RSU of an intersection per moment. In order to design TSC, the estimated delay time should be accumulated for specific period. With this processing, two indicators can be determined, accumulated delay time and accumulated vehicle sensed time. The division of which, give a percentage delay indicator for the vehicles per edge. With this indicator a lone, i.e., delay percent, the traffic conditions can be estimated but not evaluated. In order to do that, i.e., traffic conditions evaluation, evaluation period time is required. The logical relation between traffic conditions estimation and evaluation can be used directly to make a TSC adaptive. Two logical approaches can express this relation, simple and fuzzy logic. The details of these logical approaches with traffic delay estimation is going to be explained in the following subsections.

### 3.1. Traffic Delay Estimation

In PPR-based approach, PR is neither known nor can be estimated for near future. Instead of that, average speed of vehicles per edge and its derivative can be used to estimate the delay time for each incoming and outgoing edge of an intersection, as well as for the whole intersection, per moment. Estimating the delay time is not just for traffic condition estimation but also gives an indicator for evaluation period for the intersection as a whole. In general, delay time can be defined as the sum of acceleration, deceleration and stopped delay time. Where acceleration time can be defined as the time that determined with low speed and acceleration for vehicle (or group of vehicles) entered to the edge under the RSU coverage area. While, deceleration time can be defined as the time that determined with high speed and deceleration for vehicle (or group of vehicles) entered to the edge under the RSU coverage area. And finally, stopped delay time can be defined as the time that determined with zero speed and zero acceleration/deceleration for vehicle (or group of vehicles) entered to the edge under the RSU coverage area. This will be done in the RSU instantaneously per moment so that the delay time per moment can be determined. Accumulative delay time can be determined as a good indicator for evaluating TSC continually.

For each moment, the information of the group is sent to the RSU that joins the intersection. The RSU uses the incoming information for estimating the delay time and accumulated it for each edge separately. By averaging the accumulative delay time for all the edges of the intersection continuously, delay time for the whole intersection can be estimated. As the vehicle travels along an intersection encounters different degrees of delay, so the value of the average accumulative delay time varies accordingly. Intuitively, the higher value of the average accumulative delay time indicates the worse degree of traffic condition. Each RSU implementing our solution estimates accumulative delay time based on its average speed of vehicles and their derivative (acceleration/deceleration). The average speed of vehicles per edge can be obtained from V2X protocol described in [1]. Therefore, in each edge accumulative delay time in terms of its average speed of vehicles and their derivative can be estimated through fuzzy delay estimation system.

**Figure 1:**
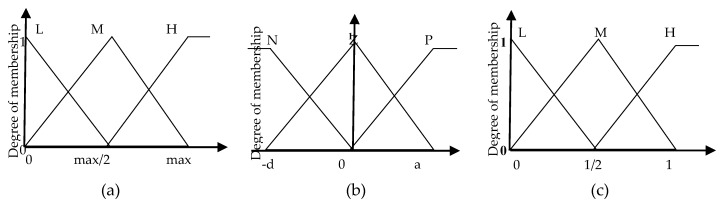
Fuzzy delay estimation system. (**a**) Average speed (m/s/veh) input sets. (**b**) Average speed derivative (m/s^2^/veh) input sets. (**c**) Average acceleration, deceleration and stopped delay (s/veh) output sets.

For fuzzy system, input variables are first classified into categories or fuzzy sets. The possible fuzzy sets for average speed are L for low, M for medium and H for high. For average speed derivative, the defined fuzzy sets are N for negative, Z for zero and P for positive. In addition, output fuzzy sets corresponding to estimated average delay time have also been defined for one second time span, with L for low, M for medium and H for high. One particularity of fuzzy logic is that a fuzzy set can contain elements with partial degree of membership and consequently an input value can belong to several fuzzy sets at the same time. For example, an average speed value of 7.5 m/s (with maximum edge speed equal to 13.889 m/s) could be member with a different degree of membership, i.e., of both medium and high average speed fuzzy sets. In order to determine the degree of membership of the input values to each of the fuzzy sets, simple membership functions are employed. Here, the membership functions used, which have been implemented based on simple rating system, are illustrated in Figure 1.

Fuzzy rules have been established as shown in Table 1 that relate input (average speed and its derivative) and output fuzzy sets (components of delay time per second).

The summation of the estimated acceleration, deceleration and stopped delay time from fuzzy system respectively, Delay(k) can be given by the following equation:

(1) Delay(k)=Acceleration(k)+Deceleration(k)+Stopped(k)

As described previously, every RSU in an intersection continuously monitors the individual estimated delay time for each edge per moment (e.g., per second) through fuzzy system. A key aspect in our traffic condition estimation is to identify the accumulative delay time close to the RSU of the intersection that will continue for specific time with suitable adaptation procedure. Adaptation procedure given here is open for further optimization. An edge is considered to have increase value of accumulative delay time if its previous estimation reported some stayed vehicle from previous cycle and such cycle is not operated well to clear all the vehicles. Every edge has a counter that represent accumulative delay time updated to the current moment according to the following equation:

(2) $$AD_{e}\left(k\right)=\{\begin{array}{cc} AD_{e}\left(k-1\right)+Delay_{e}\left(k\right) & if\;car_{e}\left(k\right)>0\;and\;k\neq 0\\ 0 & otherwise \end{array}$$

where $AD_{e}\left(k\right)$ and $AD_{e}\left(k-1\right)$ are the new and previous value of the accumulative delay time for edge e at time (k) and (k−1) respectively. $Delay_{e}\left(k\right)$ is the delay time estimated using the fuzzy system for edge e. Finally, $car_{e}\left(k\right)$ is the number of vehicles in edge e at time (k).

In order to determine the intersection delay time for all the incoming and all the outgoing edges separately, the individual $AD_{e}\left(k\right)$ for each direction are averaged as given in the following equations:

$$AD_{e}\left(k\right)=\{\begin{array}{cc} AD_{in}\left(k\right) & for\;incoming\;edges\\ AD_{out}\left(k\right) & for\;outgiong\;edges \end{array}$$

(3) $$=\{\begin{array}{cc} 1/n\sum_{e=1}^{n}AD_{e}\left(k\right) & for\;n\;incoming\;edges\\ 1/m\sum_{e=1}^{m}AD_{e}\left(k\right) & for\;m\;outgiong\;edges \end{array}$$

where *n* and *m* is number of incoming and outgoing edges with sensed vehicles respectively.

The above equations determined for each incoming and outgoing edge of an intersection. Hence, intersection delay time AD(k) as a whole can be determined by the following equation:

(4) $$AD\left(k\right)=AD_{in}\left(k\right)+AD_{out}\left(k\right)$$

Now, TSC algorithm based on traffic delay estimation can be design with acceptable and logical evaluation approach. The details of which is given in the following subsections.

### 3.2. Simple Logic TSC

As seen in COLOMBO’s TSC section, different TSC methods (i.e., policies) are developed and each one performs under specific traffic condition. Choosing suitable policy should be done in logical way rather than in an optimization way as done in COLOMBO’s solution. With simple logic approach, TSC based on traffic delay time estimation is driven here to make TSC work with different policies through policy selection procedure. Traffic delay estimation based on threshold values can be extracted directly from level of service (LOS) criteria. 

At signalized intersections, the LOS is a simple grading function (from A to F) of the average delay time [13] as shown in Table 2. It may be calculated per intersection, per edge, or per lane group.

One issue should be mentioned here, the traffic delay estimation is done instantaneously based on delay time estimation for each edge as well as for the whole intersection. The policy selection procedure of the TSC for the whole intersection should be insensitive to very short peaks delay time estimation, e.g., a singular platoon in one edge. It should react to more persistent traffic conditions changes when traffic from a single direction will continue for specific period (e.g., fifteen to twenty minutes) is expected.

In order to do that, evaluation time interval Ev(k) should be considered by the following equation:

(5) $$Ev\left(k\right)=\{\begin{array}{cc} k-LastPolicyUpdate & if\;Policy\;Changed\\ 0 & otherwise \end{array}$$

where k is the current time in second and LastPolicyUpdate is the last time in which policy had been changed.

Another important issue should be taken into consideration with low PR, several different policies can be implemented. The main difference between them is the release method that decides the actual stage duration for green light. This method returning Boolean decision result (i.e., true) when the current stage should be finished and the sequence stage should be activated based on threshold. This threshold is defined in COLOMBO project for each policy released method by multiplying the number of vehicles for each incoming lane that is currently red by the time they have been waiting (called car times second).

With low PR, number of vehicles is effected, new proposed threshold value can be used, known as accumulated delay percent ADP. It is defined as the ratio of accumulated delay time to the total vehicles sensed period. Total vehicles sensed time can be calculated by the following equation:

(6) $$Time_{e}\left(k\right)=\{\begin{array}{cc} Time_{e}\left(k-1\right)+1 & if\;car_{e}\left(k\right)>0\;and\;k\neq 0\\ 0 & otherwise \end{array}$$

From which, $ADP_{e}\left(k\right)\%$ can be determined using the following equation:

(7) $$ADP_{e}\left(k\right)\%=\{\begin{array}{cc} AD_{e}\left(k\right)/Time_{e}\left(k\right) & if\;car_{e}\left(k\right)>0\;and\;k\neq 0\\ 0 & otherwise \end{array}$$

Only when this value, i.e., $ADP_{e}\left(k\right)\%$ exceeds a predefined threshold value (e.g., 10%), RSU activate suitable control action (i.e., termination of the current green stage or not) as given by the following equation:

(8) $$Terminate=\{\begin{array}{cc} Yes & if\;\max{\left\{ADP_{e}\left(k\right)\right\}}_{e=1}^{n}>10\%\\ No & otherwise \end{array}$$

where Terminate is the activation Boolean result for termination of the current green stage to start the subsequent stage. $\max{\left\{ADP_{e}\left(k\right)\right\}}_{e=1}^{n}$ is the maximum $ADP_{e}\left(k\right)$ for n incoming edges of an intersection. After finishing all red stage, policy selection procedure should be activated based on delay time estimated for the intersection as a whole.

Each policy differs from others by the estimated traffic conditions to adjust the green time. Conditions based on predefined threshold value (e.g., average delay time and/or evaluation time interval) may be corresponds to LOS to be monitored for each edge or for the intersection as a whole.

The policy selection procedure is based on comparisons which are occurred when the group leader updates the fused data and sends it to the corresponding RSU. These comparisons are employed based on existing of vehicles and accumulative delay time estimated by different edges. In addition, the total delay time of last updating is exchanged to quantify the level of service for the intersection as a whole. With predefined threshold value, TSC policies can be changed in an adaptive way. Finally, RSU situated in the center of an intersection will get a global and complete vision of the level of delay time for the intersection as a whole as well as for each edge in the intersection.

Based on COLOMBO’s solution [14], the following conclusions can be summarized:

— Platoon and phase policies are suitable for medium and high traffic conditions.
— Platoon and swarm are suitable for very high traffic conditions.
— Marching policy is selected when the incoming edges are congested and there is no other information available about vehicles in the outgoing edges.
— Cgestion policy is selected when the outgoing edges are congested and there are vehicles waiting in the incoming edges.

If the evaluation process continues for a certain evaluation period of time (typically 15 min) with acceptable $ADP_{e}\left(k\right)$ (e.g., <10% with A LOS required), no change for intersection policy will required, otherwise policy selection procedure is activated to select new policy. With an acceptable threshold value for $AD_{e}\left(k\right)\;and\;Ev\left(k\right)$ (e.g., 35 s/veh and 900 s respectively), the following simple selection rules can be driven as shown in Table 3.

Since it is not preferred to work with specific threshold values, e.g., $AD_{e}\left(k\right)$ with 35 s/veh is suited under marching policy while $AD_{e}\left(k\right)$ with 35.1 s/veh is suited under phase policy, as well as for evaluation period, the above description for policy selection procedure can be done using fuzzy logic as described in the following subsection.

### 3.3. Fuzzy Logic TSC

**Figure 2:**
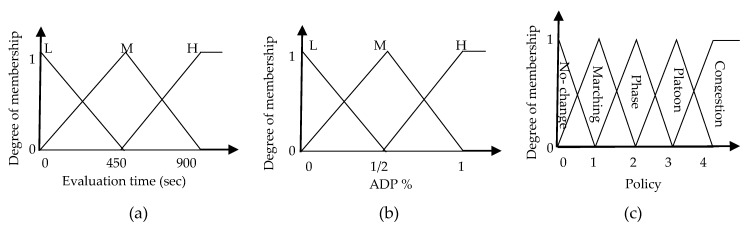
Fuzzy p system. (**a**) Evaluation time (sec). (**b**) ADP %. (**c**) Policy.

Selecting policy based on simple logic with threshold values is not preferred with estimated measurements. Because of that, another proposed logic is considered here. Beside the major measurement of delay time, evaluation time should be take into consideration. Evaluation time is the time required to evaluate each policy. Most of the standards manuals, e.g., [15], typically used 15–20 min to evaluate TSC. This value may not be reached with low PR, though our fuzzy TSC will considered the evaluation time as the second input beside accumulated delay percent $AD_{e}\left(k\right)\%$. The possible fuzzy sets for the evaluation time are L for low, M for medium and H for high. For the $AD_{e}\left(k\right)\%$, the defined fuzzy sets are also L for low, M for medium and H for high. In addition, output fuzzy sets corresponding to policy output have also been defined as no-change, marching, phase, platoon and congestion. The membership functions used in our TSC, based on simple rating system, are illustrated in Figure 2a–c.

Fuzzy rules that relate input fuzzy sets (accumulated delay percent ADP(k)% and evaluation time Ev(k) and output fuzzy set (policy) have been established as shown in Table 4. The fuzzy rules have been designed based on logical input/output relationship. As Figure 2c illustrates, the output of the fuzzy TSC system (i.e., policy selection procedure) is a continuous value within the interval [0 4] indicating the policies options.

Simulations have been run on Lenovo laptop with Core i5 processor under Linux operating system and results of the current proposed approaches are described in the following section. All figures are drawn in Microsoft Excel 2013.

## 4. Results

**Figure 3:**
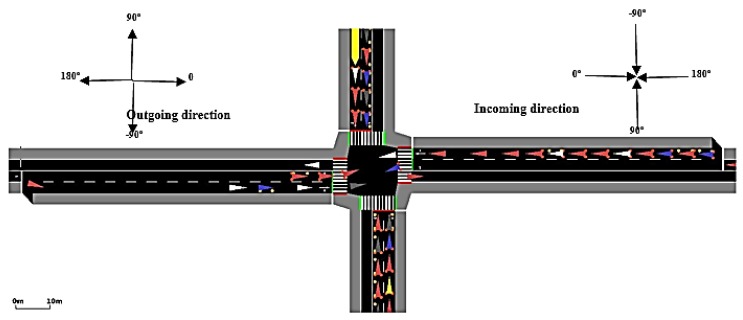
RILSA Intersection with incoming and outgoing direction.

For evaluating the proposed TSC approach, a simple scenario consisting of an intersection was taken from COLOMBO framework (called Rilsa intersection [15], as shown in Figure 3). This is done for two reason, comparability and traffic realistic. 

At COLOMBO framework, communications using IEEE 802.11p with ETSI ITS G5 standards are performed by the ns-3 standard yans Wi-Fi model. A 6 Mbps bandwidth rate with OFDM. To compute signal loss default log-distance propagation model is used. COLOMBO framework use SUMO as the traffic simulator for urban mobility to supply simulation environment. All simulations were performed in the same one hour time span. Table 5 reports the main parameters and configurations used in our simulations.

**Figure 4:**
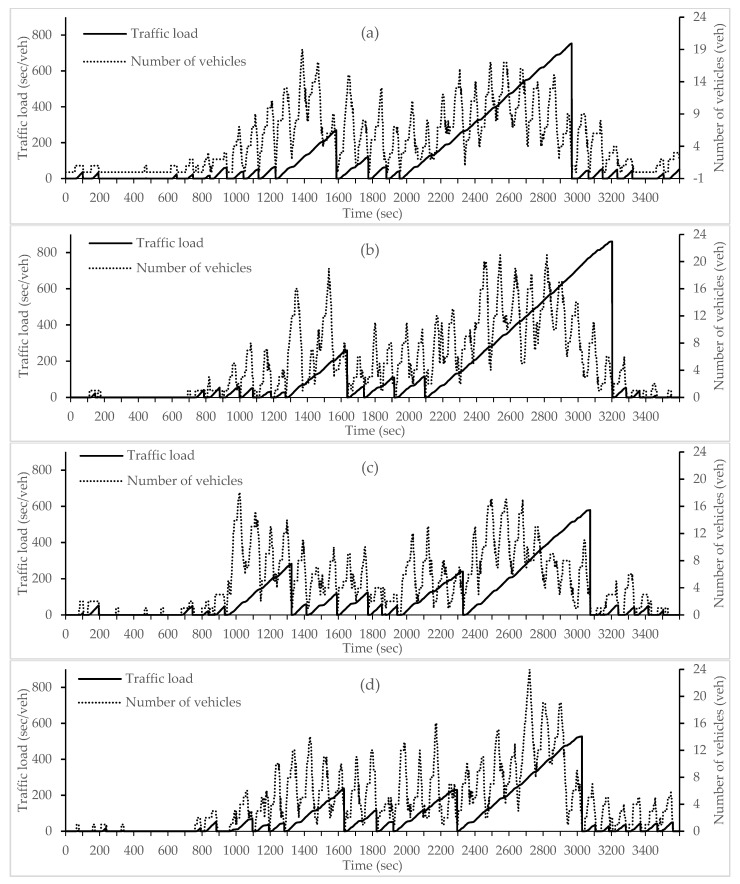
Traffic load with number of vehicles per incoming edge (**a**) 180°, (**b**) 90°, (**c**) 0° and (**d**) −90° direction respectively.

**Figure 5:**
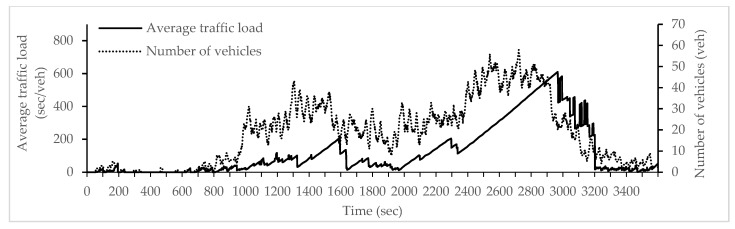
Average traffic load with number of vehicles for all the incoming edges of an intersection.

Vehicle densities and traffic conditions change dynamically with time according to a wave trend. This wave trend follows the green and red timings controlled by the traffic signals. Thus, the applied traffic conditions are dynamically generated with COLOMBO framework according to this wave trend. In spite of that an approximate number of vehicles can be specified (i.e., around 1450 vehicle) as well as various segments with different traffic loads can be stated here (Figure 4 and Figure 5 show that and for more information please see [15]).

**Figure 6:**
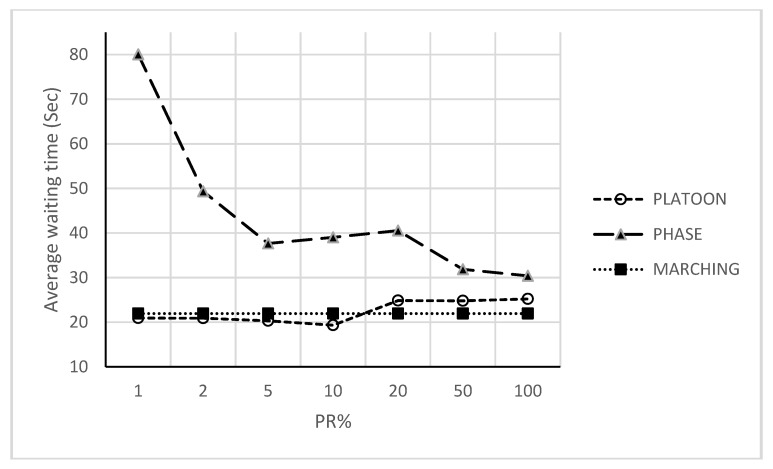
Average waiting time versus penetration rates with each policy alone.

First, simulations have been run using one single policy at a time to compare with. Simulated policies involved the following: marching, platoon, phase and congestion. Then, COLOMBO framework with swarm, simple and fuzzy logic algorithms are simulated. All of the above simulations had been run for measuring how the average waiting time varies depending on the PR. Average waiting time depicts the number of steps in which the vehicle speed was below 0.1 m/s measured in simulation steps from SUMO output. Figure 6 show the first step for each policy (i.e., phase, platoon and marching) under different PR. 

**Figure 7:**
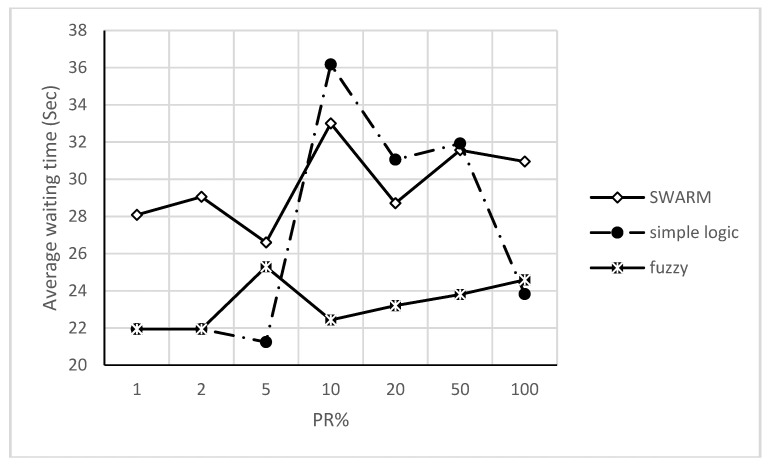
Average waiting time versus penetration rates with policy selection procedure algorithms.

Figure 7 shows the second step for COLOMBO framework with swarm, simple and fuzzy logic algorithms under different PR. These figures (i.e., Figure 6 and Figure 7) show policies behavior when simulated each one alone as well as with swarm, simple and fuzzy logic algorithms in COLOMBO framework under different PR. To evaluate our approach and compare it with the over mentioned ones, a simple comparison can be made as shown in the following section.

## 5. Comparison

In order to evaluate our approach, a simple comparison with COLOMBO approach is done in this section. Since COLOMBO project investigates this comparison with the previous researches [16], the same thing can be done here with our approach. 

Figure 6 shows that marching policy works well for all PR. This policy adopts a static approach (i.e., constant TSC setting) with dynamic phase selection. Since RILSA intersection simulated using only two phases (i.e., no need for dynamic phase selection) and using COLOMBO framework (i.e., with optimized parameters), this may reflect some sort of optimized results with this policy.

Phase policy fits well for low PR while it does not for high PR. The green light in this policy maintains since no cars on the other directions are sensed. This behavior is suitable for a dominant traffic flow (i.e., high traffic flow even under low PR) opposed by an irregular one.

**Figure 8:**
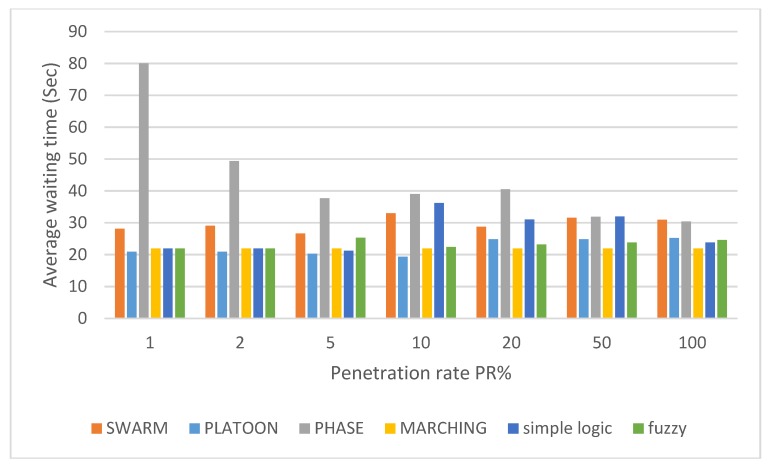
Average waiting time versus penetration rates.

Platoon policy gets significant results at low-medium PR (i.e., 2–20%) and minimum values at medium PR (i.e., 50%). As long as nothing blocks the vehicles after passing the central area, this policy creates platoons of vehicles that are free for leaving the intersection. Creating a platoon requires some characteristics, such as platoon size, length and period, to be available to study their behavior. Such characteristics are difficult to study especially under PPR approach for TSC adaptive problem. The above results clearly indicate that PPR approach should be handled under specific policies. Best TSC approach should be able to properly detect each policy situation and Figure 8 shows all of them under different PR. Swarm algorithm (more specifically, policy selection procedure of it) select between the above policies with dynamic mechanism. This mechanism depends on parameters optimization that provide smooth transitions between policies. In spite of that, this transition has a clear oscillation effect with PR > 10%. In other words, swarm algorithm has an optimization solution with unstable transition effect rather than direct policy selection procedure. On the other hand, using simple and direct if-then rules (with fuzzy delay estimation) give the same behavior as for individual policies based on threshold values for policy selection procedure. Both of the above policy selection procedures give comparable results with different PR except at 10%.

Finally, the simulated approaches combined in Figure 8 to give clear comparison between them. The results of our proposal TSC show the capability to maintain almost the same performance versus different PR of equipped vehicles. These comparable results led us to the following section of conclusion.

## 6. Conclusions

From the previous comparable results, some conclusions can be stated. Our TSC solution includes several simplifications compared to COLOMBO’s one, taking into consideration PPR based approach. The policy selection procedure in COLOMBO’s solution is not relevant for the calculation of TSC setting with low PR because it is based on counting vehicles. This can be a serious issue if not all vehicles are sensible like in FPR based approach. That’s why different versions of policy selection procedure had been proposed as an optimization problem in COLOMBO’s solutions. On the other hand, our solution has a very positive logical behavior of policy selection procedure. The TSC evaluation strongly depends on the policy selection procedure. Using threshold values (based on LOS criteria), as in our solution for policy selection procedure, makes it open for further optimization. With these threshold values our solution can get almost the same performance even when one vehicle is sensed under the RSU communication range without relying on counting vehicle. This motivates the solution to be used for PPR based approach. At the same time, focusing on developing policy selection procedure with threshold values based on delay time estimation is not enough. 

In fact, as shown in Figure 5, the duration for each vehicle to accomplish their route gives an indicator for another parameter that should be taken into consideration: evaluation period. Most TSCs are evaluated for typically 15 min as stated earlier. But none of the compared approaches for policy selection procedure had been taken this period into consideration. The lack of data for intervals where no equipped vehicle was sensed is an obvious issue. Having no data for an interval depends on PR as well as the aggregation interval’s duration. Low PRs as a result show data lacks at times where no equipped vehicle has been within the communication range. One disadvantage of our proposal is how to overcome longer time periods of TSC operation with no equipped vehicle. This had been considered in COLOMBO project but not in ours. Hence, our approach should be further investigated by taking evaluation period into consideration.

## Figures and Tables

**Table 1:** Fuzzy rules relating inputs (average speed and its derivative) with outputs (average acceleration, deceleration and stopped delay time).

Average Acceleration, Deceleration and Stopped Delay Time		Average Speed Derivative		
		N	Z	P
**Average Speed**	**L**	L,H,L	L,L,H	H,L,L
	**M**	L,M,L	L,L,M	M,L,L
	**H**	L,L,L	L,L,L	L,L,L

**Table 2:** LOS criteria.

LOS	Control Delay (sec/veh)
A	≤10
B	10–20
C	20–35
D	35–55
E	55–80
F	>80

**Table 3:** Simple logic rules for policy selection of TSC.

If	Ev(k)	And	ADP(k)	then	Policy
1	<900	=	-		NoChange
2	≥900	=	≤10	=	Marching
3	=	=	10−20	=	Marching
4	=	=	20−35	=	Marching
5	=	=	35−55	Phasing
6	=	=	55−80	=	Platoon
7	=	=	>80	=	Congestion

**Table 4:** Fuzzy logic rules for policy selection of TSC.

Plicy		Evaluation Time Ev(k) (s)		
		L	M	H
**Accumulated delay percent **ADP(k)%	**L**	NoChange	NoChange	NoChange
	**M**	NoChange	Marching	Marching
	**H**	Phase	atoon	Congestion

**Table 5:** Simulation parameters.

Parameter	Value
Wi-Fi mode	802.11p/ETSI ITS 5G
Transmission mode	6 Mbps (OFDM)
Node radius	170 m
Propagation loss	Logarithmic
Propagation speed	Constant (3 × 10^8^ m/s)
Penetration rate	100, 50, 20, 10, 5, 2, 1%
Simulation time	1 h

## Nomenclature

TSC	Traffic signal control
V2V	Vehicle to vehicle
V2I	Vehicle to infrastructure
V2X	Vehicle to everything
L	Low
M	Medium
H	High
Z	Zero
VANET	Vehicle ad hoc network
RSU	Road side unit
FPR	Full penetration rate
PPR	Partial penetration rate
VTL	Virtual traffic light
